# Editorial: Network Communication in the Brain

**DOI:** 10.1162/netn_e_00167

**Published:** 2020-11-01

**Authors:** Daniel Graham, Andrea Avena-Koenigsberger, Bratislav Mišić

**Affiliations:** Department of Psychology, Hobart and William Smith Colleges, Geneva, NY, USA; University Information Technology Services, Indiana University, Bloomington, IN, USA; McConnell Brain Imaging Centre, Montréal Neurological Institute, McGill University, Montréal, Quebec, Canada

**Keywords:** Brain connectivity, Communication models, Connectome, Network dynamics, Controllability

## Abstract

Communication models describe the flow of signals among nodes of a network. In neural systems, communication models are increasingly applied to investigate network dynamics across the whole brain, with the ultimate aim to understand how signal flow gives rise to brain function. Communication models range from diffusion-like processes to those related to infectious disease transmission and those inspired by engineered communication systems like the internet. This Focus Feature brings together novel investigations of a diverse range of mechanisms and strategies that could shape communication in mammal whole-brain networks.

How does a massive network of neurons give rise to intercommunication across the entire brain? As advances are made in understanding mammalian brain network structure (see, e.g., Assaf et al., 2020; Bassett & Sporns, 2017), the question of how such networked neural elements intercommunicate, and ultimately give rise to brain function, is undoubtedly one of the most intriguing scientific inquiries today (Avena-Koenigsberger et al., 2018).

Being the pinnacle of complex systems, brain networks can be studied across a spectrum of spatial and temporal scales that span various orders of magnitude. On one end of the scale, single-neuron biophysical models only partially constrain the range of possible solutions as to how communication takes place (and single-neuron models are themselves undergoing revision, e.g., Sardi et al., 2017; Gidon et al., 2019). On the other end, at the whole-brain level, emergent network dynamics could resemble physical processes such as diffusion or driven dynamical systems, but could also resemble dynamics of infectious diseases, engineered communication networks like the internet, or other systems. This Focus Feature investigates a diverse range of mechanisms and strategies that could influence communication across mammal whole-brain networks.

Leading off is compelling evidence from Seguin et al. (2020) as to the importance of including communication goals and constraints in modeling brain network dynamics. Using human structural and functional imaging data, Seguin et al. show that existing approaches based on network structure alone predict little variance in node activity. In contrast, approaches that include an explicit model of network communication perform substantially better. Seguin et al. find that the best predictors assume signals are communicated on random walks or on short paths determined from a node’s knowledge of local network structure. Simulated activity under these approaches also performs almost as well as empirical functional activity in predicting behavioral dimensions of individuals, such as tobacco use.

The realization that many complex networks share common architectural traits and statistical properties has afforded network neuroscience a broader perspective of communication models that can be implemented to study brain networks. Three papers in this Focus Feature examine mechanisms that are novel or that are applied in a new context.

Lella et al. (2020) use a model of network communication inspired by infectious disease spreading to illuminate Alzheimer’s disease (AD). They construct an analytical model of network-wide communication from structural imaging of healthy humans and those with AD. The model assumes that nodes can utilize redundant paths, a property that is quantified via a measure termed *communicability* (see Estrada & Hatano, 2008; Crofts & Higham, 2009). This measure shows much greater differences between patient and control participants compared to a shortest path measure. Strikingly, and counterintuitively, the model shows that nodes in the AD brain are actually closer to one another in terms of the communicability measure, presumably due to the pattern of network damage engendered by the disease. The authors conclude that this result supports the notion that AD is spread along brain networks via an infectious disease vector. This work connects with studies of similar types of communication models that are increasingly used to understand the spread of misfolded proteins across a range of neurodegenerative diseases (for a review, see Carbonell et al., 2018).

Shadi et al. (2020) test a thresholding model on the mouse connectome, based on an elaboration of influences of single-neuron dynamics. Their model, termed an asynchronous linear threshold model (Granovetter, 1978; Mišić et al., 2015), includes a McCulloch–Pitts-like threshold based on empirical connection weights (tracer-based fiber volumes) in combination with a consideration of empirical physical distances between nodes and resultant signal delays. The behavior of this intriguing model suggests that a few regions such as the claustrum and posterior parietal cortex are instrumental in generating cascades of multimodal sensory signals that ultimately spread throughout the brain.

A crucial component of brain network dynamics that has received little attention concerns interactions among signals. Most models assume signals do not interact (but see Mišić et al., 2014). However, Hao and Graham (2020) argue that interactions are likely, given the extremely short network distances between nodes in mammal brain networks. Hao and Graham (2020) focus on collisions, which are ubiquitous in large-scale engineered communication systems. They compare numerical simulations of two routing protocols when collisions are considered: a standard random walk strategy and an “information spreading” scheme similar to the infectious disease model of Lella et al. (2020). In simulations on two tracer-based connectomes of the macaque monkey cortex and one of the mouse whole brain, Hao and Graham (2020) show that information spreading actually achieves lower overall activity and greater sparseness of activity compared to a random walk model. Hao and Graham (2020) provide evidence that the mammal brain network is well suited to generating efficient network communication through a dynamic interplay of signal creation and destruction.

The hierarchical nature of brain networks also likely influences communication strategies (Vázquez-Rodríguez et al., 2019). Vázquez-Rodríguez et al. (2020) investigate how hierarchies within and across modalities guide network communication based on imaging data. Their analysis shows that messages are likely to be passed to nodes nearby in the hierarchy. Furthermore, they begin to broach the question of selective control of signals, which must operate on brain networks given that network structure is fixed over the short term but yet must achieve real-time routing of attention, decision outputs, invariances, etc. (see Graham & Rockmore, 2011). Vázquez-Rodríguez et al. (2020) demonstrate the potential for systematic “detours” or re-routing of messages, especially in attentional networks, which could achieve selectivity.

One of the main assumptions underlying these studies is that the network organization, beyond its individual components, is what largely conditions the emergence of higher level communication dynamics. In other words, these models do not rely on top-down control of signal flow in the brain (as there is in traditional telephone networks, for example). However, this does not mean that a subset of nodes cannot exert strong influence on the entire network. The Focus Feature concludes with two papers that consider the role of controllability, a notion borrowed from the study of dynamical systems in physics. Controllability in networks captures the degree to which network dynamics can be driven by a small subset of nodes.

Patankar et al. (2020) examine the relationship between network structure and controllability. Utilizing numerical simulations over networks derived from human structural imaging data, they show that the relationship between network structure and controllability is complex, and that it depends in part on connection weights. Specifically, Patankar et al. (2020) find that measures of network modularity are not closely related to controllability, whereas measures that consider connection weights and hub-like properties can succeed in predicting controllability.

Finally, Srivastava et al. (2020) provide a review of control theory as applied to brain networks, focusing on similarities and differences between frameworks based on network control and those based on network-wide communication. Srivastava et al. (2020) show intricate connections and contrasts related to the models’ level of abstraction, dynamical complexity, and other factors. They argue that the two frameworks can and should be integrated to build richer and more insightful models of whole-brain dynamics.

This collection of studies broadens the range of communication models in brain networks and highlights novel structural and functional demands that are likely at play. One can expect that this work will lead to further blossoming in this area of investigation and a deeper consideration of network communication in other areas of brain science.
